# Adaptive reuse in the healthcare industry: repurposing abandoned buildings to serve medical missions

**DOI:** 10.1186/s12913-017-2339-4

**Published:** 2017-07-11

**Authors:** James K. Elrod, John L. Fortenberry

**Affiliations:** 1 Willis-Knighton Health System, 2600 Greenwood Road, Shreveport, LA 71103 USA; 2 LSU Shreveport, 1 University Place, Shreveport, LA 71115 USA

**Keywords:** Adaptive reuse, Repurposing, Growth strategy, Spatial expansion, Healthcare

## Abstract

**Background:**

Adaptive reuse—the practice of identifying, acquiring, renovating, and placing back into service a building or similar structure for a purpose different than that for which it was originally designed—offers great potential for addressing the spatial expansion needs of healthcare establishments in a unique and mutually beneficial manner. This repurposing approach, however, has received very little attention in the health sciences literature, diminishing the opportunities of those serving in hospitals, medical clinics, and related care providing institutions to acquire an understanding of the practice.

**Discussion:**

The delivery of healthcare services primarily is site based, requiring physical space for physicians, nurses, administrators, and others to carry out the many duties associated with the provision of medical care and attention. But this space often represents a significant expenditure, consuming financial resources which otherwise could be directed toward patient care. Economies on this front are possible through adaptive reuse, permitting more resources to be directed toward mission fulfillment activities. This article directs attention toward adaptive reuse by profiling Willis-Knighton Health System’s associated experiences and implementation strategies. Among other things, opportunities and obstacles are discussed, detailed cases are presented, and an operational framework is provided, permitting healthcare providers to understand and make use of this novel practice for addressing spatial expansion needs more affordably.

**Conclusions:**

Since space considerations exist throughout the lives of healthcare establishments, providers must ensure an awareness of methods for productively attending to these requirements. Evidenced by Willis-Knighton Health System’s associated experiences and outcomes, adaptive reuse presents an option for more economically addressing spatial requirements, fostering opportunities to expand the delivery of health and medical services.

## Background

Despite advancements in virtual, mobile, and related technologies, the delivery of healthcare services remains largely site based, requiring physical space for physicians, nurses, administrators, and others to conduct all of the activities associated with the provision of medical care and attention. Space considerations exist very obviously for new healthcare establishments just opening their doors to patients, but they remain concerns even as institutions mature, especially in cases where patient volume outstrips capacity, necessitating additional space, and in situations where expansion initiatives take healthcare organizations into new communities.

Often times, this space, whether owned or leased, carries significant costs, reducing financial resources that otherwise could be directed toward patient care [1, 2]. Real estate expenditures can be so pronounced that they can dissuade or even prohibit expansion, diminishing abilities for healthcare providers to deliver more care to more people. Quite obviously, achieving economies on this front can permit more resources to be directed toward mission fulfillment, namely delivering health and medical services to patients.

One method for achieving such economies rests with a particular approach known as adaptive reuse. This practice entails the identification, acquisition, and renovation of existing, abandoned buildings, placing them back into service on behalf of their new owners and reinstating them as assets, rather than blights, in their given communities. Adaptive reuse has been profiled extensively in many industries, with retail, hospitality, and housing sectors being notable examples [3–5]. The healthcare industry has received some attention (e.g., [6–10]), but it has been very sparse, diminishing the opportunities of those serving in hospitals, medical clinics, and related care providing institutions to acquire an understanding of the practice.

This article directs attention toward adaptive reuse by profiling Willis-Knighton Health System’s associated experiences and implementation strategies. Among other things, opportunities and obstacles are discussed, detailed cases are presented, and an operational framework is supplied, permitting healthcare providers to understand and make use of this novel practice for addressing spatial expansion needs more affordably, fostering opportunities to expand the delivery of health and medical services.

### Definition

Adaptive reuse is defined as the practice of identifying, acquiring, renovating, and placing back into service a building or similar structure for a purpose different than that for which it was originally designed. Sometimes termed repurposing, the technique often centers on breathing new life into abandoned buildings in various states of decline. Although it contains a significant renovation component, adaptive reuse is much broader due to its focus on modifying structures to accommodate new and different missions. It’s been shown to help communities maintain or recapture vitality, reduce blight caused by abandoned properties, and conserve natural resources, all while permitting institutions to economically address their spatial expansion needs [11–13].

Abandoned buildings emerge for myriad reasons ranging from progress, which compels establishments to relocate to better serve clients, to misfortune, which prompts businesses to cease operations at particular locations. Regardless of cause, vacant properties remain, representing unrealized potential. Adaptive reuse allows enterprising institutions to tap into this potential, addressing spatial expansion requirements and restoring to service an otherwise unproductive property [14]. One institution possessing extensive adaptive reuse experience is Willis-Knighton Health System.

### Willis-Knighton Health System and adaptive reuse

Willis-Knighton Health System is a nongovernmental, not-for-profit healthcare provider delivering comprehensive health and wellness services through multiple hospitals, numerous general and specialty medical clinics, an all-inclusive retirement community, and more. Based in Shreveport, Louisiana, the system holds market leadership in its served region, centered in the heart of an area known as the Ark-La-Tex, where the states of Arkansas, Louisiana, and Texas converge.

Willis-Knighton Health System’s experience with adaptive reuse began decades ago. The system’s origins date to 1924 with the establishment of Tri-State Sanitarium, founded to address the healthcare needs of the burgeoning population of west Shreveport. Sold in 1929 to Drs. James Willis and Joseph Knighton, the establishment continued operations and, in 1952, it was renamed in honor of Drs. Willis and Knighton.

For the first several decades of its existence, the establishment played an important but relatively small role in delivering the region’s healthcare. In the 1970s, however, Willis-Knighton Health System embarked on a detailed growth campaign to expand its footprint beyond west Shreveport. With funding to support growth initiatives being in short supply, Willis-Knighton Health System was forced to economize, leading executives to consider repurposing existing, abandoned structures to support growth ambitions.

Initial and subsequent adaptive reuse experiences proved economical and effective, resulting in the practice becoming a deeply ingrained part of Willis-Knighton Health System’s culture. With over 20 adaptive reuse projects having been accomplished successfully, it’s the first consideration whenever facing expansion needs that cannot be accommodated by renovating existing space. New construction is pursued only after adaptive reuse possibilities have been investigated and determined to either be nonexistent or unviable. The practice is considered to be a core component of Willis-Knighton Health System’s overall strategy due to its history of enhancing the institution’s competitive advantages.

### Selected examples

Reviewing several examples profiling Willis-Knighton Health System’s repurposing initiatives perhaps is the best starting point for developing an understanding of adaptive reuse. The variety evident in the noted examples illustrates the depth and breadth of adaptive reuse possibilities, some being obvious and others less so.

#### WK Innovation Center (formerly Bossier Medical Center)

Built in 1966, Bossier Medical Center provided healthcare services for many years to the population of Bossier Parish, Louisiana before closing in the 1990s. Subsequent providers occupied the facility until the early 2000s when the property was abandoned. Unoccupied and exposed to the elements, the building fell into disrepair, blighting one of the busiest transit corridors in the community. In 2012, desiring a location to house a consolidated business office, centrally and securely store medical records, house system-wide education initiatives (e.g., WK’s virtual hospital, new employee orientation center), showcase its legacy (e.g., WK’s Talbot Medical Museum), and provide a conference venue for internal and community use, Willis-Knighton Health System canvassed the marketplace to identify potential repurposing opportunities. The former Bossier Medical Center emerged as a top candidate.

Ensuing assessments prompted purchase of the property after which it was comprehensively revitalized for its new role, emerging in 2015 as the WK Innovation Center. Repurposing the 185,000 square foot property cost $16 million (acquisition plus renovations), reflecting a $26 million savings compared to the $42 million estimate for equivalent new construction. Willis-Knighton Health System fulfilled its spatial expansion needs at a bargain price and the community received a new, state-of-the-art facility in place of urban blight.

#### WK Rehabilitation Institute (formerly Doctors’ Hospital)

Once a cornerstone of healthcare, Doctors’ Hospital of Shreveport closed permanently in 2010, bringing to an end a legacy which began in 1907. The idled building’s appearance declined greatly in forthcoming years due to inactivity and exposure, but its solid structure and especially its ideal location, overlooking the heart of downtown Shreveport, made it an attractive adaptive reuse candidate.

In 2015, seeking a location to house its center of excellence for rehabilitative care, Willis-Knighton Health System purchased the building, comprehensively repurposing it to serve this new and different healthcare mission. Modifications even included reorienting the building’s main entrance to face the primary transit corridor leading to the facility, improving its roadside presence and enhancing access for patients. Completed in 2017 and reintroduced as the WK Rehabilitation Institute, the 150,000 square foot building cost $26 million (acquisition plus renovations), a bargain price, especially when considering that equivalent new construction would total $44.5 million.

**Fig. 1:**
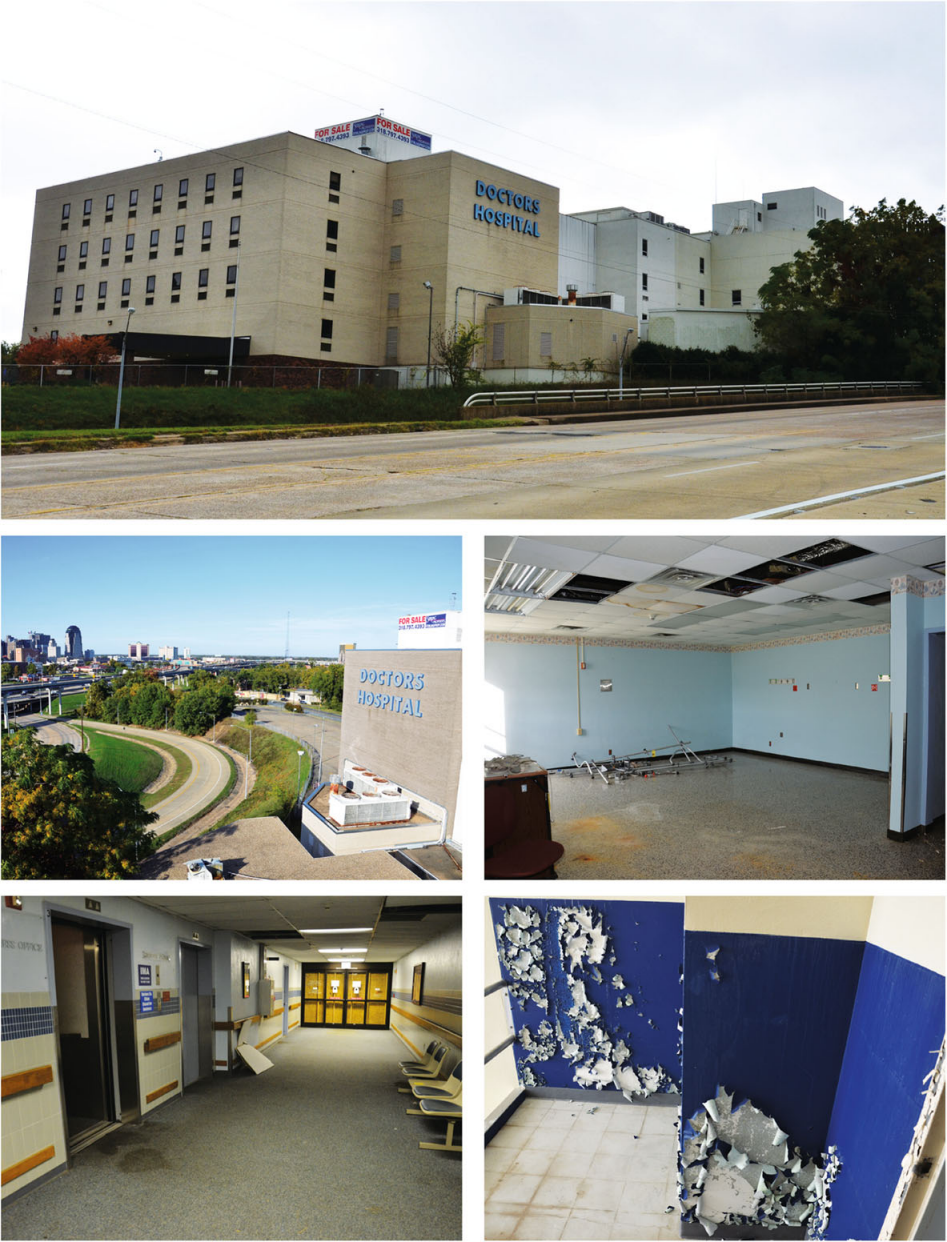
Doctors’ Hospital, as it appeared before Willis-Knighton Health System’s acquisition. Copyright © 2014 Willis-Knighton Health System. Used with permission. The featured photographs present Doctors’ Hospital of Shreveport just before its acquisition by Willis-Knighton Health System in 2015. Complete with a “For Sale” sign atop its tower and razor wire barriers around its perimeter, the dilapidated facility appeared well suited only for the wrecking ball. Willis-Knighton Health System, however, realized the building’s potential and on acquisition began concerted adaptive reuse efforts to effect its transformation

**Fig. 2:**
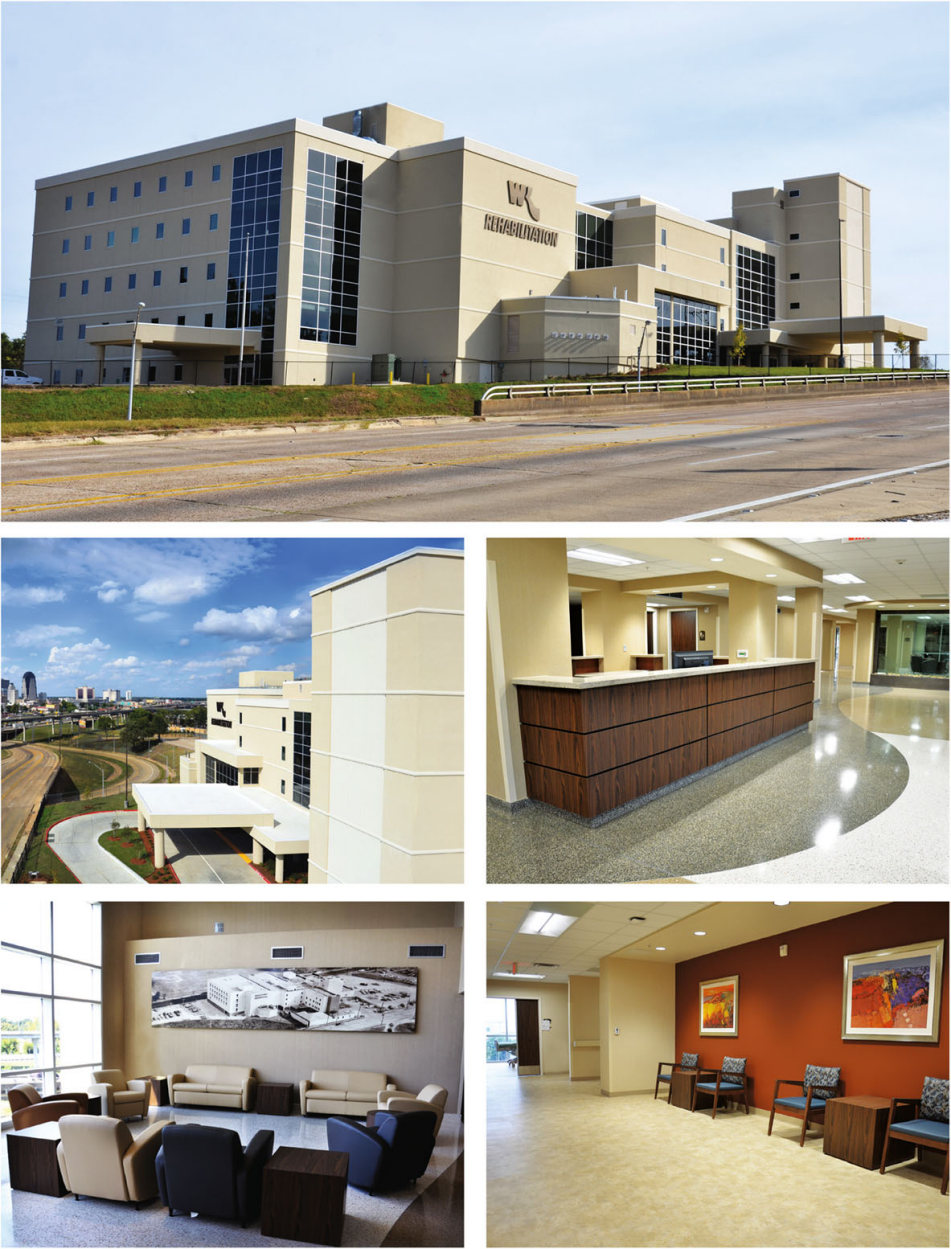
The WK Rehabilitation Institute, repurposed from Doctors’ Hospital. Copyright © 2016 Willis-Knighton Health System. Used with permission. The dramatic adaptive reuse transformation of the former Doctors’ Hospital of Shreveport into Willis-Knighton Health System’s newest center of excellence, the WK Rehabilitation Institute, is clearly presented in the noted photographs. The resulting building, reintroduced in 2017, is virtually indistinguishable from brand new construction and features an entirely revised facade, all-new interior elements, and an enhanced layout which facilitates access. A property that once blighted the community now serves as a symbol of innovation and progress

Beyond an $18.5 million savings, adaptive reuse afforded Willis-Knighton Health System with one of Shreveport’s most iconic locations and gave the city a virtually brand new institution providing an impressive entryway into downtown. The resulting transformation, illustrated in Figs. 1 and 2, has been welcomed by governmental officials and other stakeholders for its positive impact on the aesthetics and economics of Shreveport.

#### WK Portico Medical Mall (formerly Portico Shopping Center)

A retail shopping center, anchored by a Brookshire’s Food and Pharmacy grocery store, offered another attractive adaptive reuse candidate for Willis-Knighton Health System. Situated across the street from one of the system’s hospitals, WK Pierremont Health Center, the shopping center, constructed in 1992, was acquired in 1999 with plans to use the available space to address capacity limitations experienced at the neighboring hospital.

Comprehensively revitalized and reintroduced as the WK Portico Medical Mall, the 136,000 square foot establishment houses the system’s center of excellence for orthopedic care, physician offices, and a range of support services. The $13 million project (acquisition plus renovations) afforded a significant discount compared to the new construction estimate of $41 million, yielding a $28 million savings thanks to the economies offered by adaptive reuse.

#### WK Fleet Service Center (formerly Gulf Oil Service Station)

Seeking a place to provide routine maintenance for its growing fleet of service vehicles, Willis-Knighton Health System investigated options and discovered an opportunity adjacent to its main campus in an available Gulf Oil service station. The owners of the 1950s–era service station desired to exit the business in the late 1980s and sought a buyer. After learning of Willis-Knighton Health System’s interest, the owners ultimately decided to donate the property to the system, affording them tax benefits associated with supplying a gift to a charitable institution.

Repurposing was quick and easy, as the nearly 3000 square foot service station already contained vehicle service bays, hydraulic lifts, and additional equipment needed for the building’s revised role of maintaining the system’s many vehicles. Additional modifications were necessary to convert the premises to serve an institutional mission, but these were minimal. The service station reemerged in 1990 as the WK Fleet Service Center, carrying a total cost of $50,000, a significant savings compared to the new construction estimate of $360,000, afforded by the generosity of the service station’s owners and the economies of adaptive reuse. It now services a fleet of over 120 vehicles, including company cars, delivery trucks, and patient transportation vans.

#### WK Palmetto Center (formerly Palmetto Country Club)

Willis-Knighton Health System’s latest adaptive reuse project is ongoing and entails the conversion of the recently closed Palmetto Country Club into a comprehensive healthcare campus. Founded in 1950, the country club closed in 2014 due to declining memberships. It featured an 18-hole golf course, club house, and swimming pool situated on a 155-acre site in Benton, Louisiana. Willis-Knighton Health System had been monitoring this high-growth marketplace for an attractive expansion opportunity and, after careful consideration, decided to purchase the property which over the course of a decade will emerge as a full campus. The first step, currently underway, involves repurposing the existing clubhouse and swimming pool to serve as a WK Fitness and Wellness Center. Preliminary analyses suggest that Benton, Louisiana will need comprehensive acute and long term care services in coming years. Service lines will gradually be introduced at WK Palmetto Center as community need dictates.

Due to the ongoing nature of the project, ultimate savings have yet to be determined, but the country club was acquired at a cost considered to represent an exceptional value. The majority of the resulting campus indeed will require new construction, but the existing infrastructure formerly used by the country club is being incorporated into the revised plans in full, demonstrating that adaptive reuse can be deployed in combination with new construction, conserving resources and bolstering associated value.

### Opportunities

Willis-Knighton Health System’s adaptive reuse experiences suggest that the practice affords a range of opportunities, most of which are unavailable through any other spatial expansion method. The observed attributes are supported in the literature of adaptive reuse and they also confirm the notion that adaptive reuse delivers a wealth of mutual benefits.

#### Financial incentives

Adaptive reuse has the potential to deliver significant financial savings unavailable through equivalent new construction projects [15]. This benefit, observed in each of Willis-Knighton Health System’s experiences, is cited as the primary motivation for pursuing adaptive reuse [14–16]. The economies associated with the practice primarily result from the highly favorable pricing of properties which effectively have concluded their first lives and no longer are able to attract tenants. Buildings that are unoccupied, especially for lengthy periods of time, often present opportunities for negotiating attractive pricing and sometimes feature discounted pricing at the onset [11, 13, 14].

Additional savings are possible by reusing some or all of the existing building’s infrastructure in the revised property, reducing building materials and labor costs [17–20]. Loans and grants tied to historic preservation, redevelopment, and related initiatives might also be available, offering an additional opportunity to achieve economies [14, 16].

The savings potential of adaptive reuse certainly shouldn’t be lost on those serving in the healthcare industry. Just as Willis-Knighton Health System discovered in its initial growth endeavors, adaptive reuse, courtesy of the savings generated, can make possible spatial expansion which wouldn’t be feasible under new construction scenarios. It also helps demonstrate fiscal responsibility, something especially important for government healthcare institutions and not-for-profit establishments, as they face the heightened expectations of the citizenry to use resources judiciously. Further, the economies derived from adaptive reuse can facilitate institutional efforts to deliver charitable care to underserved populations. Willis-Knighton Health System’s indigent clinic network, for example, has made use of the practice, permitting an increasing percentage of charitable dollars to be directed toward care provision in these needy communities. When viewed in this light, adaptive reuse has deeper strategic implications than might be assumed at face value.

#### Premium location availabilities

Many variables converge to create an idyllic location for healthcare operations, especially those involving care delivery which must ensure safe, convenient access for patients. Often, the best locations in communities are already occupied, but relocations or closures can once again free them up, albeit with existing structures onsite. Through adaptive reuse, these highly-prized locations can be brought back into consideration [11, 12].

In each of Willis-Knighton Health System’s adaptive reuse experiences, the locations acquired were deemed to be excellent. Several properties, in fact, afforded one-of-a-kind visibility and access in their given markets.

#### Community renewal support

Abandoned buildings represent intensive problems for communities. Over time, they can become blights, which diminish scenic beauty. Safety hazards also emerge, resulting from inactivity which leaves abandoned structures vulnerable to criminal and environmental elements. Further, tax revenues are lost. The opportunity to return an abandoned building to productive service facilitates community renewal efforts, eliminating blight, fostering job creation, and generating goodwill that garners the support of political leaders, neighboring businesses, and the citizenry [11, 13, 14].

Through its adaptive reuse endeavors, Willis-Knighton Health System has endeared itself to stakeholders ranging from top elected officials to private citizens who appreciate the renewal afforded by the system’s investments. While healthcare institutions have long sought to be excellent partners with their given communities, usually via traditional routes such as offering vital medical services, adaptive reuse provides a pathway for enhancing the resulting partnerships through contributions that far exceed healthcare delivery.

#### Conservation of resources

Adaptive reuse supports many conservation efforts, thus bolstering the environmental stewardship credentials of those healthcare institutions that turn to the practice. By repurposing an existing, abandoned building, its destruction can be averted, reducing the burden on landfills which otherwise would have to accommodate the waste [21–23]. Further, even when adaptive reuse projects require extensive renovations, the natural resource requirements are fewer than those associated with new construction, representing a more eco-friendly option [19, 20]. As an environmentally responsible institution, Willis-Knighton Health System strives to maximize opportunities for conservation and adaptive reuse clearly has fostered associated efforts.

### Obstacles

Healthcare institutions desirous of pursuing adaptive reuse should be aware of obstacles that might potentially be encountered. These sometimes present insurmountable challenges and other times they require additional effort to overcome, with each case being situation dependent.

#### Insufficient or nonexistent availabilities

A very obvious obstacle to adaptive reuse simply entails the lack of a suitable and available building in a desired area. Available structures must possess a range of qualities, determined by the prospective buyer, to be viable adaptive reuse candidates. Suitable candidates may or may not exist, something that can only be determined through exploration and investigation [14, 24].

#### Excessive renovation costs

Even when a suitable prospective building is identified meeting location, size, and other desirables, on closer inspection, the structure might be determined to be too expensive to renovate. While modern design and construction techniques can make outdated structures new again, meeting even the loftiest standards for patient care delivery, limits indeed can be reached after which the associated costs of the renovation outweigh the benefits. Damage from things such as flooding, fire, vandalism, and environmental contamination can be so severe that repairs would not be economically feasible. Ultimately, the magnitude of negative encumbrances in the context of the prospective buyer’s tolerance regarding the cost of remedies will determine whether the site remains an option or should be discarded in favor of other opportunities [14, 19, 20].

#### Disagreements with stakeholders over reuse

In some cases, establishments might be forced to forego an adaptive reuse candidate because community stakeholders find the prospective reuse to be undesirable. Occasionally, this dissatisfaction is expressed through public protests and related conveyances. Adaptive reuse by definition entails a building reemerging to serve a purpose different than the one pursued originally, setting the stage for at least some to be disenchanted. Much depends on the nature of the new use and whether those in the community view it to be a benefit or detriment to the given locale [13, 25].

When evaluating candidate properties, Willis-Knighton Health System proactively engages public officials, community leaders, and private citizens, informing them of envisioned plans for the rebirth of the given structure. Feedback is sought, and if possible, stakeholder suggestions are incorporated into associated plans. This approach ensures that envisioned plans are communicated accurately. It also helps to gauge stakeholder interest in the proposed project, providing a key indicator for ascertaining if the pursuit is worthwhile.

#### Zoning difficulties

Since adaptive reuse implies that an existing building will reemerge to serve a new purpose different from that of its original mission, zoning and related regulatory matters must be taken into consideration when evaluating any candidate property. Converting a property from one classification to another may or may not be possible, with this being dependent on local rules and regulations governing commercial operations [14, 16, 20]. Quite obviously, such matters must be thoroughly investigated well in advance of purchase, making sure that any regulatory issues encountered can be traversed successfully.

### Operationalization

Willis-Knighton Health System’s methods for operationalizing adaptive reuse evolved from its own experiences. Lacking the benefit of an underlying knowledge base available in the health sciences literature, executives were forced to rely on intuition, creative thinking, and a willingness to experiment and learn from successes and failures. With each adaptive reuse experience, expertise increased and eventually led to the operational plans that remain in use today.

Since adaptive reuse is merely an option for consideration whenever spatial expansion needs present themselves, simple awareness of the practice is the initiating operational requirement. An awareness of adaptive reuse places it into the realm of possibilities for addressing space requirements, but a formal protocol is needed to ensure its consideration. The usual and customary process associated with realizing a spatial expansion involves seven stages as follows.

1. Needs assessment
2. Site selection and acquisition
3. Concept development and approval
4. Design development and approval
5. Construction, completion, and inspection
6. Commercial preparation
7. Grand opening

During Stage 2, site selection and acquisition, opportunities emerge to consider adaptive reuse candidates. To formalize consideration, Willis-Knighton Health System devised a 4-step decision-making model, termed the Adaptive Reuse Consideration Framework, placing it within the site selection and acquisition stage. Illustrated in Table 1, the framework is explained as follows.

**Table 1:** The adaptive reuse consideration framework

Step 1. market surveillance *Before considering new construction, investigate the availability of an adaptive reuse candidate property* Action steps: a. Canvass desirable locations for candidate properties b. Enlist the assistance of a real estate professional to aid in the search *Is an adaptive reuse candidate available? If YES, proceed to Step 2. If NO, consider new construction.*
Step 2. preliminary analysis *Conduct a preliminary analysis of the adaptive reuse candidate property* Action steps: a. Assemble a team consisting of key personnel involved in the expansion initiative b. Visit the site and perform a walk-through inside and around the candidate property c. Hold a “reimagination session” to envision possibilities *Are the results of the preliminary analysis satisfactory? If YES, proceed to Step 3. If NO, consider new construction.*
Step 3. feasibility study *Conduct a feasibility study of the adaptive reuse candidate property* Action steps: a. Perform a customer service assessment Example considerations: i. Is the size of the structure adequate to serve the targeted customer base? If not, are renovations possible? ii. Does the location facilitate pedestrian and vehicular customer traffic (e.g., safety, accessibility, parking)? iii. Does a good fit exist between the candidate property and existing system components, permitting cohesion in service delivery? b. Perform a political assessment Example considerations: i. Will the intended use of the property require zoning changes or other regulatory considerations? ii. Are public officials supportive of the intended use of the property? iii. Is any discontent anticipated among the citizenry regarding the intended use of the property? c. Perform a financial assessment Example considerations: i. Does the cost of the project (property cost plus required renovations) make economic sense? ii. What savings, incentives, or other financial benefits, if any, are anticipated to be afforded over an equivalent new construction scenario? iii. Are any other realistic scenarios possible which might yield a better financial result? *Are the results of the feasibility study satisfactory? If YES, proceed to Step 4. If NO, consider new construction.*
Step 4. property acquisition *Acquire the property and proceed to the concept development and approval stage*

### Step 1: market surveillance

When facing a need for physical space that cannot be met by renovating existing infrastructure, instead of immediately focusing on acquiring a vacant parcel of land upon which to place new construction, executives first scout the general area where the expansion is needed to determine if an abandoned and available building that can accommodate the desired application exists. This is a fairly quick process, as availabilities can easily be ascertained either by personal site visits to canvass the area or by requesting the assistance of commercial real estate professionals with firsthand knowledge of availabilities.

If availabilities do not exist, then adaptive reuse pursuits are abandoned, with traditional new construction pathways being pursued instead. However, if a building with the potential to be repurposed exists, executives conduct a preliminary analysis to determine its suitability.

### Step 2: preliminary analysis

The preliminary analysis begins with a site visit conducted at the candidate property by an evaluation team consisting of executives, key personnel who will be responsible for overseeing the forthcoming operation, engineering and construction personnel, and architecture and design consultants. The site visit permits team members to view the current state of the premises and ascertain its potential to be repurposed. Importantly, this comprehensive tour provides a context for reimagining the structure as a new and improved entity fulfilling the mission called for by the expansion. If the structure is deemed to be worthy of accommodating the reimagined application, a deeper feasibility study is conducted.

### Step 3: feasibility study

The feasibility study examines the merit of the candidate property on customer service, political, and financial fronts. The customer service assessment seeks to determine if the prospective property can adequately accommodate target audiences. Among other things, efforts are initiated to determine if the size of the structure is appropriate for the given application and, if not, what degree of renovation would be necessary to make the accommodation. Pedestrian and vehicular traffic patterns are studied to ensure safe and convenient access for those who will be using the envisioned facility. Vehicle parking requirements also are investigated. Further, efforts are taken to determine if cohesion between the new location and the greater institution will be sufficient to ensure that support services can be delivered in a problem free manner.

The political assessment primarily focuses on zoning and associated regulatory issues concerning the property, investigating what steps, if any, are needed to permit a healthcare-related mission to take place at the noted site. Additionally, efforts are directed toward ascertaining the degree of stakeholder support associated with the intended mission.

The financial assessment investigates the cost of the prospective project (acquisition plus renovations) in an effort to determine whether the pursuit makes economic sense. Resulting figures then are compared with estimates of the cost of an equivalent new construction project, giving executives a useful evaluative reference. Attention also is directed toward ascertaining what, if any, alternative scenarios are possible and, if so, what are their associated costs, providing yet another comparison to help ensure a financially prudent decision.

The details and insights gained from the customer service, political, and financial assessments are compiled and presented in ensuing meetings to render a decision regarding feasibility. If obstacles which cannot be overcome are revealed in the findings, barring the emergence of another prospective adaptive reuse candidate, a new construction scenario will be pursued.

### Step 4: property acquisition

If the adaptive reuse candidate is deemed to be meritorious, the property will be acquired and the effort will advance to the concept development and approval stage. The reimagined site will be formalized by commissioned designers, architects, and engineers, eventually resulting in a completed, repurposed building that satisfies the institution’s expansion needs and once again delivers value in the community.

### Addressing technical complexities

Willis-Knighton Health System’s Adaptive Reuse Consideration Framework provides a basic overview of the steps required to evaluate adaptive reuse candidates and its use helps to ensure that the practice is considered when spatial expansion decisions are at hand. Due to the inherent complexities associated with repurposing abandoned buildings to serve medical missions, it goes without saying that the application of this framework requires healthcare institutions to direct significant time and attention toward each step. While the range of associated tasks might seem overwhelming, perhaps to the point of discouraging some from pursuing adaptive reuse, Willis-Knighton Health System has observed from its experiences that relying on qualified and capable experts reduces burdens significantly.

Willis-Knighton Health System’s approach for ascertaining the financial feasibility of an adaptive reuse candidate generally begins by calculating the total cost per square foot for an equivalent new construction project using historical knowledge and industry standards. This provides a rough estimate of the institution’s cost ceiling for the given project, offering a working measure with which to compare the potential savings associated with the adaptive reuse candidate under review. If this appears promising, executives then arrange for formal cost estimates to be prepared by architecture and engineering firms, ultimately affording an accurate adaptive reuse versus new construction cost comparison which Willis-Knighton Health System’s executives can use to make an informed decision.

Similarly, engineering and construction concerns associated with adaptive reuse candidates (e.g., physical plant assessments, hazardous waste identification and removal issues, code compliance matters) are evaluated with the assistance of professional firms. These entities possess the expertise required to make proper assessments, supply associated advice, and issue reliable cost estimates, further shoring up information needs.

Scores of decisions indeed are required throughout the Adaptive Reuse Consideration Framework. While some can be addressed internally with relative ease (e.g., composition of evaluation teams, savings percentages required to select adaptive reuse over new construction, etc.), others call for expertise that might not be available within given healthcare establishments. In such cases, knowledge gaps can be filled with the assistance of external parties, making pursuit of adaptive reuse candidates possible, even for healthcare institutions with little internal expertise on building and construction fronts.

## Conclusions

Evidenced by Willis-Knighton Health System’s associated experiences, adaptive reuse offers a unique and mutually beneficial method for addressing the spatial expansion needs of healthcare institutions, providing those serving in hospitals, medical clinics, and other care providing facilities with an option to consider beyond traditional renovation and new construction pathways. The practice by its very nature coincides nicely with the community-minded, altruistic missions typically espoused by healthcare establishments, affording an exceptional strategic fit when proper circumstances present themselves. While adaptive reuse has received very little attention in the health sciences literature, through the repurposing insights and operational guidance supplied in this article, it is hoped that knowledge and awareness of the practice will be bolstered, permitting healthcare providers to understand and make use of this novel method for addressing spatial expansion needs more affordably, fostering opportunities to expand the delivery of health and medical services.
